# KOC is a novel molecular indicator of malignancy

**DOI:** 10.1038/sj.bjc.6600790

**Published:** 2003-03-04

**Authors:** F Mueller, M Bommer, U Lacher, C Ruhland, V Stagge, G Adler, T M Gress, T Seufferlein

**Affiliations:** 1 Division of Gastroenterology, Department of Medicine, University of Ulm, Germany; 2 Division of Hematology/Oncology, University of Ulm, Germany

**Keywords:** KOC, tumour marker, fine-needle aspiration

## Abstract

The detection of malignant cells in fine-needle aspirates (FNA's) using marker genes is hampered by the fact that these markers are only expressed by certain malignancies or lack sensitivity and/or specificity. Here we report the results of a prospective pilot study examining the expression of KOC (KH-domain containing protein over expressed in cancer), a novel onco-foetal gene, in 76 patients who underwent fine-needle aspiration for further diagnosis of abdominal lesions, aszites, cysts or cerebrospinal fluid. Aspirates were examined by cytology and by a KOC RT–PCR assay. KOC expression was a highly sensitive and specific indicator of malignancy. The KOC assay could be useful to facilitate screening for malignant disease and to improve the diagnostic accuracy of FNAs.

In a large-scale screen for differentially expressed genes in pancreatic cancer, we have recently identified a gene encoding a novel protein with four K-homologous (KH) domains, which is highly overexpressed in pancreatic cancer (Mueller-Pillasch *et al*, 1997). The new gene was named KOC (***K****H*-domain containing protein ***o****verexpressed* in ***c****ancer*). Two known functions of KH-domain containing proteins are the regulation of mRNA stability and subcellular localisation, both of which are implicated in fundamental biological processes such as development, cell growth, differentiation and carcinogenesis (Ross *et al*, 1997). Therefore, KOC may play a role in the regulation of tumour cell proliferation by interfering with transcriptional and/or post-transcriptional processes. However, the precise role of KOC in tumour biology remains to be elucidated. Our preliminary data suggested that KOC is exclusively expressed in tumours and embryonic tissues (Mueller-Pillasch *et al*, 1997,^1999^) and may therefore represent an ideal target for novel diagnostic approaches. To test this hypothesis, a prospective study was undertaken to determine the diagnostic accuracy of measuring KOC expression as compared to the cytological assessment in a consecutive series of fine-needle aspirates (FNAs) from abdominal lesions, aszites, various cysts and cerebrospinal fluid.

## MATERIALS AND METHODS

### Determination of KOC expression in cell lines and FNAs

Human Panc-1 pancreatic cancer cells were purchased from the American Type Culture Collection. Stocks were maintained in Dulbecco's modified Eagle's medium (DMEM) supplemented with 10% (v v^−1^) foetal bovine serum (FBS) in a humidified atmosphere of 5% CO_2_/95% air at 37°C and passaged every 3 days. Confluent cultures of Panc-1 cells were trypsinised and resuspended in serum-free DMEM. Various numbers of Panc-1 cells were subsequently mixed with 1 ml of human serum and then immediately centrifuged at 1200 r.p.m. for 2 min. The supernatants were decanted. The remaining cell pellets were resuspended in the RNeasy Mini Kit lysis buffer (Qiagen, Hilden, Germany) and total RNA was extracted using the same kit according to the manufacturer's instructions. cDNA synthesis and PCR were performed with the one-step RT–PCR (Polymerase Chain Reaction) Kit (Qiagen, Hilden, Germany) using KOC gene-specific primers. Annealing temperature was 55°C for 32 cycles. PCR products were run on a 1% agarose/TAE gel and stained with ethidium bromide. Diagnostic FNAs of various abdominal lesions or fluids were obtained by ultrasonography-guided biopsy using a 0.7 mm Chiba needle. The first blowout of the needle was used for cytology. Cells remaining in the needle were flushed into a vial with sodium chloride and subsequently further analysed for KOC expression as described above. Informed consent of all patients was obtained prior to the procedure. The KOC RT–PCR assays and the cytological examination were performed independently in a blinded fashion by different researchers. The diagnostic sensitivity of cytology and the KOC RT–PCR assay was determined as the frequency of samples correctly identified as malignant/positive by each examination among the number of correctly identified positive cases plus the number of false-negative cases. The diagnostic specificity of cytology and the KOC RT–PCR assay was determined as the frequency of samples correctly identified as benign/negative by each examination among the number of correctly identified negative cases plus the number of false-positive cases.

## RESULTS AND DISCUSSION

**Figure 1:**
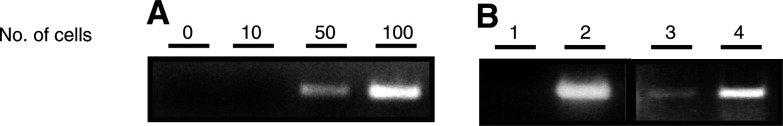
Expression of KOC in pancreatic cancer cells and tissue samples of various cancers. (**A**) Various numbers of Panc-1 cells were mixed with 1 ml of human serum and then immediately centrifuged at 1200 r.p.m. for 2 min. Total RNA was extracted and KOC expression was determined by RT–PCR as described in Materials and Methods. (**B**) Typical results of KOC RT–PCR assays performed as described above using RNA extracted from residual cells in the aspiration needle after FNA: **1**=normal pancreas, **2**=metastasis of colorectal cancer, **3**=lymphoma, **4**=hepatocellular carcinoma. Samples 2–4 are classified as KOC positive.

To determine the number of cells required to detect KOC transcripts, various numbers of Panc-1 human pancreatic cancer cells, which express KOC (Mueller-Pillasch *et al*, 1997), were mixed with 1 ml of human serum. As few as 50 cancer cells were sufficient to demonstrate KOC transcripts in this cell line (Figure 1A

).

The first blowout containing the majority of the aspirate was always used for the cytological assessment to assure optimal conditions for cytology. The second blowout was used to determine KOC expression. Using this approach KOC transcripts were clearly detectable in aspirates of various malignant lesions (Figure 1B).

Having established that KOC transcripts could be detected under these conditions, aspirates of 48 patients (20 women and 28 men, median age of 62.5 and 59.7 years, respectively; range 45–77 and 27–82 years, respectively) who underwent FNA for further diagnosis of an abdominal lesion (median size 4.4 cm; range 1.1–10 cm) were examined by cytology and the KOC RT–PCR assay. A definitive diagnosis could be obtained in 41 patients by cytology or by additional histological examination of specimens obtained by trucut biopsy or surgical procedures. In another seven patients, the initial cytological assessment was indeterminate, but these patients were not available for a further FNA. Thus, those 41 cases with a definitive cytological assessment and/or histological confirmation were enrolled in the study.

By initial cytological analysis, 24 out of 41 FNAs were diagnosed as malignant (Table 1A

**Table 1:** (A) Summary of the results of the initial cytological and subsequent histological analysis of repeat biopsies and the KOC RT–PCR assay in FNA samples^a^ and >(B) Clinical characteristics of patients undergoing FNA

**(A)**						
**No. of patients**	**Cytology**		**Repeat biopsies**	**KOC**		**Confirmed diagnosis Malignant/benign**
23	+			+		23/0
1	+			−		1/0
2	?		+	+		2/0
1	?	−		+		0/1
1	?		−	−		0/1
4	−		+		+	4/0
9	−			−		0/9
T=41						
**(B)**						
**No. of patients**	**Localisation of lesion**	**Cyto/Histo +**	**KOC +**	**Cyto/Histo −**	**KOC −**	**Confirmed diagnosis**
3	Liver	3	3			Colorectal cancer
1	Spleen	1	1			Colorectal cancer
1	Retrocaval lymph node	1	1			Colorectal cancer
1	Liver	1	1			Pancreatic cancer
5	Pancreas	6	6			Pancreatic cancer
7	Liver	7	6		1	Cancer of unknown primary
4	Truncal lymph node	3	4	1		Lymphoma
2	Liver	2	2			Hepatocellular carcinoma
1	Peripancreatic lymph node	1	1			Plasmocytoma
1	Spleen	1	1			Lung cancer
1	Liver	1	1			Breast cancer
1	Liver	1	1			Gastric cancer
1	Pelvis	1	1			Ovarian cancer
1	Pancreas	1	1			Renal cancer
4	Liver			4	4	Not malignant disease
3	Pancreas			2	2	Not malignant disease
3	Mediastine			3	3	Not malignant disease
1	Pelvis			1	1	Not malignant disease
						
T=41		30	30	11	11	

a +=expression of KOC or lesion classified as malignant in cytology and/or histology; −=no expression of KOC detectable or lesion classified as benign in cytology and/or histology. ?=lesion classified as indeterminate in cytology. T=total.

). A total of 23 of these samples were also KOC positive. Only in one sample KOC transcripts could not be detected. Four lesions were classified as indeterminate by cytology. In two of these lesions malignant disease was subsequently detected on repeat biopsy. In these two lesions, KOC transcripts were already detected in the first FNA. Another two of the initially indeterminate lesions were subsequently classified as benign on repeat biopsy. One of these lesions was KOC negative in the first FNA. However, KOC transcripts were detected in the other lesion. In total, 13 samples were initially classified as benign by cytology. However, in four of these patients, malignant disease was strongly suspected and ultimately confirmed on repeat biopsy. All four samples were KOC positive already in the first assessment. Another nine lesions were negative by both cytology as well as the KOC assay. The sites of abdominal lesions and the confirmed diagnoses are shown in Table 1B.

Diagnostic sensitivity and specificity of the KOC assay in FNAs were 93% (29 out of 31) and 83% (10 out of 12), respectively.

The detection of malignant cells in fluids by cytology is often difficult. Therefore, we examined whether the KOC assay could also be useful to detect malignant cells in aspirates of fluids. Aspirates included aszites, fluid from liver, pancreatic and mediastinal cysts as well as cerebrospinal fluid. In total, 50 aspirates were examined. In 35 aspirates from 35 patients, a definitive cytological statement could be obtained (13 women and 22 men, median age of 59.1 and 60.5 years, respectively; range 22–86 and 22–92 years, respectively; for patient characteristics, see Table 2

**Table 2:** Clinical characteristics of the patients whose aspirates were examined for KOC expression

**No. of patients**	**Localisation of fluid**	**Main diagnosis**	**Cyto +**	**KOC +**	**Cyto −**	**KOC −**
2	Aszites	Not malignant disease			2	2
4	Aszites	Colorectal cancer	2	2	2	2
2	Aszites	Gastric cancer			2	2
2	Aszites	Pancreatic cancer	1	1	1	1
1	Aszites	Liver cancer			1	1
1	Aszites	Liver abscess			1	1
2	Aszites	Ovarian cancer	2	2		
1	Mediastinal fluid	Lymphoma	1	1		
2	Mediastinal cyst	Mediastinal cyst			2	2
1	Mediastinal cyst	Squamous cell carcinoma			1	1
1	Liver cyst	Liver cyst			1	1
1	Pancreatic cyst	Pancreatic cyst			1	1
2	Cerebrospinal fluid	Lymphoma	2	2		
1	Cerebrospinal fluid	Leukaemia	1	1		
12	Cerebrospinal fluid	Leukaemia			12	12
T=35			9	9	26	26

+=Expression of KOC or aspirate classified as malignant in cytology; −=no expression of KOC detectable or aspirate classified as benign in cytology. T=total.

). Subsequently, these 35 aspirates were also examined for KOC expression.

In total, nine aspirates were cytologically diagnosed as malignant and 26 as benign. KOC expression was detected in all nine cytologically malignant aspirates. All 26 cytologically benign samples were also KOC negative. In particular, we obtained 15 samples of cerebrospinal fluid from patients who presented with acute leukaemia or lymphoma and underwent lumbar puncture to determine involvement of the central nervous system. Three of these samples were classified as malignant by cytology. KOC expression was detected in all three samples. In contrast, the 13 samples classified as benign by cytology exhibited no KOC transcripts (Table 2).

Thus, diagnostic sensitivity and specificity of the KOC assay in these aspirates was 100% if cytology is taken as the gold standard. The aspirates of seven patients were classified as benign by cytology and KOC negative despite the fact that these patients were diagnosed with a malignant disease. However, more invasive procedures to further investigate the existence of malignant cells in these fluids were not justified from a medical point of view.

The data of our pilot study establish a striking difference in KOC expression between malignant and benign lesions. KOC transcripts are expressed by a broad spectrum of malignant cells originating from primary epithelial tumours, metastases and even haematopoietic malignancies. KOC transcripts can be detected in samples that are readily obtainable such as FNAs and aspirates including cerebrospinal fluid with a high sensitivity and specificity.

Various screening markers have been tried to increase the diagnostic accuracy in FNAs and aspirates. However, these markers remain unsatisfactory because of their low sensitivity and/or specificity or because of their limited applicability, for example, telomerase in breast lesions (Clark *et al*, 1997) or ret/PTC rearrangements in thyroid cancer (Cheung *et al*, 2001). The KOC assay is generally applicable and could be a useful novel tool to refine the FNA diagnosis of malignancy by reducing the number of additional procedures required to establish a suspected diagnosis of malignancy. The assay facilitates the screening for malignant disease, but could also be used to monitor therapeutic efficacy, for example, in cerebrospinal fluid. Further studies are needed to determine the onset of KOC expression during carcinogenesis and to establish whether detection of KOC transcripts could be even useful as an early indicator of malignancy.
